# Physiological and perceptual demands of singles and doubles beach tennis in women of different competition levels

**DOI:** 10.3389/fphys.2024.1434636

**Published:** 2024-10-30

**Authors:** Nathalia Jung, Leandro Carpes, Lucas Domingues, Rodrigo Abreu, Magni Mohr, Rodrigo Ferrari

**Affiliations:** ^1^ Postgraduate Program in Human Movement Sciences, School of Physical Education, Universidade Federal do Rio Grande do Sul, Porto Alegre, Rio Grande do Sul, Brazil; ^2^ Sports and Exercise Training Study Group, Clinical Research Center, Hospital de Clínicas de Porto Alegre, Porto Alegre, Rio Grande do Sul, Brazil; ^3^ Postgraduate Program in Cardiology, School of Medicine, Universidade Federal do Rio Grande do Sul, Porto Alegre, Rio Grande do Sul, Brazil; ^4^ Centre of Health Sciences, Faculty of Health Science, University of the Faroe Islands, Tórshavn, Faroe Islands; ^5^ Department of Sports Science and Clinical Biomechanics, SDU Sport and Health Sciences Cluster (SHSC), Faculty of Health Sciences, University of Southern Denmark, Odense, Denmark

**Keywords:** accelerometer, exercise physiology, recreational sports, heart rate, racket sports

## Abstract

**Purpose:**

To analyze and compare the physiological responses of women during singles and doubles beach tennis sessions.

**Methods:**

In this crossover trial, twenty-two women with previous participation in amateur beach tennis tournaments randomly performed two experimental sessions: singles and doubles beach tennis. The routine for both sessions consisted of 10-min of seated rest, followed by 45-min of beach tennis and 30-min of post-exercise recovery. Participants were matched against opponents of the same competition level, defined according to their local beach tennis ranking (advanced or intermediate level). They warmed up with basic techniques for 5-min and played 3 matches lasting 12-min, interspersed with 2-min recovery intervals. Heart rate (HR), energy expenditure (EE), number of steps (STEPS), handgrip strength (HS), rating of perceived exertion (RPE), and enjoyment were assessed throughout the sessions. Generalized estimating equations were employed to examine the main effects between experimental sessions over the time and in relation to competition level.

**Results:**

HRmean and HRmax (Δ: HRmean = 13 ± 3 bpm; HRmax = 11 ± 3 bpm) as well as EE and Steps (Δ: EE = 66 ± 22 kcal; RPE = 2 ± 0 A.U.; Steps = 250 ± 52 A.U.) were higher in singles than doubles (*p* < 0.05). The percentage of total time spent in the highest HR zone (91–100%HRmax) was significantly greater in singles than in doubles (39% ± 22% vs. 15% ± 18%; *p* < 0.05). Differences were found in the percentage of total time spent in each HR zone, recovery HR, and HS between competition levels (*p* < 0.05).

**Conclusion:**

Singles beach tennis resulted in higher physiological demands than doubles in women, and players’ competition level partly affects the training responses.

## 1 Introduction

Beach tennis is a fast-growing sport with over 1 million practitioners in Brazil, regulated by the International Tennis Federation that promotes over 300 tournaments in 37 different countries (Tennis Rules and Regulations-ITF, 2024). The game is played on a sand court and is a mix of tennis and beach volleyball, as it uses racquets and lower pressurized tennis balls, however the ball is not allowed to touch the ground. A net split the court in half at 1.70 m in height. The sport can be played in doubles (2 vs. 2) or individually (1 vs. 1) in a court the same size of beach volleyball for doubles (16 m × 8 m) or smaller for singles (16 m × 4.5 m) (Tennis Rules and Regulations-ITF, 2024). Due to the characteristics of doubles beach tennis matches, in which the actions are divided with the partner, the game can result in different physiological demands when compared to singles, in which the same player hit the ball every time it crosses the net. Other factors such as training status of participants, sex, age, and previous experience in the modality may also influence the physiological demands of racket sports (Fernandez-Fernandez et al., 2009; Pluim et al., 2023). However, there is currently no published study on the physiological demands of singles and doubles beach tennis.

The practitioners of beach tennis engage in the activity for both competitive and recreational purposes (Rosa and Alvarez, 2021), with some focusing on health related benefits and others in improving game performance. Our research group recently evaluated the acute effects of a beach tennis session played in doubles on cardiovascular parameters of men and women with arterial hypertension. During the 45 min of activity, the mean reserve heart rate (HR_reserve_) was ∼60%, and a moderate rate of perceived exertion was reported during the game. Additionally, we assessed post-exercise hypotension after the beach tennis session and found a reduction in 24-h ambulatory blood pressure (systolic: ≈6 mmHg; diastolic: ≈3 mmHg). In this pioneer study, the focus was to assess benefits of the activity on blood pressure in a sample consisting of untrained participants (Carpes et al., 2021). Still, it seems relevant to investigate the demands of beach tennis in previously trained individuals regularly engaged in amateur tournaments of the sport, since the results can be used to optimize preparedness for competition or to improve health parameters.

Beach tennis appears to be an intense intermittent activity that may have some of the characteristics of hybrid sports (Batrakoulis et al., 2022) such as soccer, which has been shown at meta-analysis level to be a highly efficient training modality to improve physical fitness, as well as cardiovascular, metabolic and bone health (Milanović et al., 2015; Milanović et al., 2019; Milanović et al., 2022) in sedentary populations. Similar findings have been shown in team handball (Póvoas et al., 2018), floorball (Wikman et al., 2017) and futsal (Castagna et al., 2020). At the present, the acute effects of beach tennis were only assessed in untrained hypertensive participants, and there is no data available comparing the physiological demands of singles and doubles beach tennis. The ease of learning and low motor complexity of beach tennis have attracted a large number of middle-aged female practitioners, including those who had never previously practiced any other sport. Therefore, the evaluation of a sample composed of women is particularly important to fill the longstanding gap in sports research, which has historically concentrated on male participants, resulting in the underrepresentation of women in scientific studies. Therefore, the present study tests the primary hypothesis that beach tennis played in singles has a higher physiological loading compared to doubles in women. A secondary explorative hypothesis was that the physiological loading was dependent on the players’ competition level.

## 2 Materials and methods

### 2.1 Study design and participants

This is a crossover trial in which the participants randomly performed two experimental sessions: Singles and Doubles Beach Tennis. The randomization list consisted of random blocks of four participants and was generated by independent researchers (that did not participate in the recruitment of participants or their assignment to intervention groups) using a computer software. Participants and the research team were blinded to the randomization list until the time of assignment.

Twenty-two middle-aged women were found eligible and volunteered to participate in the study. The sample size was estimated to detect a minimum difference of 10% (8 bpm) in maximum HR during singles and doubles beach tennis sessions (Alcock and Cable, 2009). The calculation was carried out using the PSS Health tool online version 19 (Borges et al., 2020), considering a power of 80% and a significance level of 5%. The inclusion criteria were middle-aged women (35–55 years old), affiliated to the Beach Tennis Federation of Rio Grande do Sul (Brazil) with previous participation in amateur tournaments of this federation. Exclusion criteria included ischemic heart disease, angina pectoris, stroke or heart failure diagnosed in the last 24 months, and musculoskeletal problems that hinder exercising. Recruitment was carried out through telephone calls or face-to-face invitations during beach tennis tournaments.

Ethical committee approval was obtained at the Institutional Review Board of Hospital de Clínicas de Porto Alegre, Brazil (approval number: 5.309.930). The study protocol was conducted in accordance with the Declaration of Helsinki and the Brazilian resolution number 466/12 of research involving human beings. All participants provided written informed consent before entering the trial. The experimental sessions were conducted between January and December of 2022 in the city of Porto Alegre (RS, Brazil).

### 2.2 Characteristics of the experimental sessions

Participants were instructed to avoid vigorous physical activity the day before the experimental sessions, to keep their regular diet, and to not ingest alcohol, caffeine, and other stimulants on the same day of the session. All sessions started between 8:00 and 10:00 a.m. (at the same time of the day to account for potential diurnal physiological variation) and lasted approximately 2 h. The participants were hydrated at the beginning of the session and were allowed to drink water *ad libitum* to ensure the maintenance of proper hydration throughout the sessions. Temperature and relative humidity during the sessions were collected to ensure that both sessions were performed under the same environmental conditions, and the interval between the sessions was 5–7 days.

The players were blinded to their partners and opponents until the intervention day. They were assigned to the matches against other players of the same competition level, defined according to their local beach tennis ranking (defined by the Beach Tennis Federation of Rio Grande do Sul, Brazil), and confirmed by two researchers with previous experience in the modality. The Brazilian Beach Tennis Federation defines the beach tennis categories for amateur tournaments in levels A, B, C, D and beginners. “A” is the highest-level category of amateur tournaments, and “D” is the lowest level of amateurs’ players with previous experience in beach tennis. For the purpose of this study, players were classified into 2 levels: Level 1 included advanced players with ranking in A and B (Advanced level), and level 2 included participants with ranking in C and D categories (Intermediate level).

Both sessions followed the same routine that consisted of 10 min of seated rest, followed by 45 min of beach tennis and 30 min of recovery after exercise. The participants warmed up with basic techniques for 5 min (volleys and serve) and played 3 matches of 12 min each, with 2-min intervals between matches. The games were played according to ITF beach tennis rules.

### 2.3 Assessments

*Heart rate* was monitored and recorded during and up to 5 min after the end of the session using a chest monitor (Polar H10, Finland). After the match, the heart rate data was downloaded to a computer, and the average and maximal heart rate was automatically calculated using the PolarFlow software. The data were categorized into heart rate zones to indicate total time spent at ≤60, 61–70, 71–80, 81–90, 91%–100% maximal heart rate (HR_max_) using the same software. The individual HR_max_ was determined according to the following formula: 220 minus age or the highest HR value reached during the matches, if the value was higher than the estimated HR_max_. The reserve HR (HR_reserve_) was calculated using the following formula: ((exercise HR - resting HR)/reserve HR) *100 (Swain and Leutholtz, 1997).

*Energy expenditure* (EE) of the sessions was estimated based on the HR values through the PolarFlow software. The software was configured based on individual parameters including age, height, weight, maximal heart rate, and gender, enabling automatic EE calculations (Gilgen-Ammann et al., 2019).

*Number of steps* was measured during the matches using an accelerometer (GT3X - ActiGraph Inc, Pensacola, FL, United States). The equipment was placed in position by the research team at the beginning of the session (when they entered the court) and removed at the end of the match. The accelerometer was fixed at the waist, on the right iliac crest, using an elastic strap, programmed with 1-s epochs, which were then converted to 15 s, using ActiLife software (version 6.8.1; ActiGraph LLC, Pensacola, FL, United States).

*Handgrip isometric strength* was assessed before (pre) and after the sessions (post 5′ and 30′) using a hydraulic handgrip dynamometer (JAMAR® 5030J1, Sammons Preston Rolyan, Bolingbrook, IL, United States). The participants were seated in armless chairs, with their elbows flexed at a 90° angle, attached to their bodies, while their shoulders and wrists were maintained in a neutral position (Vargas-Pinilla and Rodríguez-Grande, 2021). Following instructions and a demonstration, a proficient researcher positioned in front of each participant grasped the device during the test and instructed them to exert maximum force while squeezing. This measurement procedure was repeated thrice on each arm, with a 1-min interval between readings. All participants used the same second handle position, and standardized verbal encouragement was provided during the measurements.

*Rating of perceived exertion* (RPE) was assessed after the warm-up, and immediately after each 12-min match (intra 1, intra 2, and intra 3) using the CR-10 Borg scale (Borg, 1990). Participants were previously familiarized with the use of scale in a preliminary session.

*Internal load* of participants was estimated using an additional RPE assessed 15 min after the completion of exercise (Haddad et al., 2017). Basically, the participant answered a simple question: “How was your training?” using the CR-10 Borg scale (Foster et al., 2001). A single arbitrary unit representing the magnitude of the internal training load for each session was calculated by multiplying the RPE and the session time (minutes).

*The enjoyment level* during the sessions was assessed using the Physical Activity Enjoyment Scale (PACES) (Teques et al., 2017). During the post-exercise period (15–30 min after the end of each session) the participant received an electronic questionnaire and was asked to rate the level of enjoyment based on the following question: “How do you feel at the moment about the physical activity you performed?” The questionnaire consisted of 18 items rated on a 7-point bipolar rating scale. A total of 11 items were reversed and scored. Summing the individual item scores generated an overall PACES score. This yielded a possible range of 18–126, and higher PACES scores reflect greater levels of enjoyment.

### 2.4 Statistical analyses

Data were entered in duplicate by two separate researchers. The statistician was not involved in the recruitment or assignment to the experimental sessions and was blinded to the interventions. The assumption of normality was assessed using the Shapiro-Wilk test. Results were presented as means and standard deviation (Table 1) or standard error (Table 2–4) for variables with a normal distribution. Generalized estimating equations (GEE) analyses were employed to examine the main effects between experimental sessions (2 sessions: Singles and Doubles beach tennis) over time and participant level (session*time). To compare the differences between participant competition levels, an additional GEE analysis was conducted, incorporating a new factor (level 1 vs. 2). Post-hoc comparisons were conducted using Bonferroni tests. Paired Student's t-tests were used to assess direct comparisons between Singles and Doubles sessions, and the association between HR and RPE was performed using Pearson’s correlation test. Statistical significance was set *a priori* at *p* < 0.05. All statistical analyses were performed using IBM SPSS Statistics for Windows, version 19 (IBM, Armonk, NY, United States).

**TABLE 1:** Characteristics of the participants.

Variables	Total (n = 22)	Level 1: Advanced (n = 11)	Level 2: Intermediate (n = 11)	*p*-Value
Age, years	40.7 ± 6.6	42.1 ± 6.9	39.3 ± 6.3	0.330
Weight, kg	65.1 ± 9.2	66.6 ± 9.2	63.6 ± 9.3	0.463
Height, m	1.7 ± 0.1	1.7 ± 0.1	1.7 ± 0.1	0.663
BMI, kg/m^2^	23.2 ± 2.9	23.5 ± 2.8	22.9 ± 3.3	0.632
Practice time, months	43.9 ± 24.5	51.9 ± 28.1	36 ± 18.4	0.131
Frequency, days.week^-1^	2 (2)	3 (3)	2 (2)	0.357
Time per week, hour.week^-1^	3.7 ± 2.6	4.6 ± 2.9	2.8 ± 2	0.104
Duration, min.day^-1^	91 ± 30	106 ± 28	76 ± 25	**0.015**

Values are mean ± standard deviation (SD) for parametric distribution data, and median (interquartile range) for non-parametric distribution data; BMI: body mass index; *p*-value is comparing the competition levels separately. Bold p-values indicate significant results (*p* < 0.05).

**TABLE 2:** Heart rate and rating of perceived exertion responses during singles and doubles beach tennis sessions.

Variables	Singles	Doubles	∆	*p*-Value
**Warm-up (5** **min)**				
HR	132 (125–138)	134 (124–144)	−2 (−9 to 6)	0.726
RHR	56 (50–61)	58 (50–66)	−2 (−8 to 5)	0.642
	(Moderate)	(Moderate)		
RPE	2 (1–3)	2 (2–3)	0 (−1 to 1)	0.213
	(Light)	(Light)		
**Intra session 1**				
HR	161 (156–165)	146 (140–153)	15 (9–19)	**<0.001**
RHR	80 (77–84)	68 (63–72)	12 (8–16)	**<0.001**
	(Vigorous)	(Vigorous)		
RPE	4 (3–5)	3 (2–3)	1 (1–2)	**<0.001**
	(Moderate)	(Light)		
**Intra session 2**				
HR	164 (159–169)	146 (139–154)	18 (10–23)	**<0.001**
RHR	83 (79–87)	68 (63–74)	15 (9–20)	**<0.001**
	(Vigorous)	(Vigorous)		
RPE	4 (3–5)	3 (2–3)	1 (1–2)	**<0.001**
	(Moderate)	(Light)		
**Intra session 3**				
HR	166 (161–171)	147 (140–154)	19 (11–24)	**<0.001**
RHR	85 (81–88)	69 (64–74)	16 (10–20)	**<0.001**
	(Vigorous)	(Vigorous)		
RPE	4 (3–5)	3 (2–3)	1 (1–2)	**<0.001**
	(Moderate)	(Light)		
**Total session (45** **min)**				
HR_max_	181 (151–160)	171 (151–160)	10 (5–16)	**<0.001**
HR_mean_	156 (151–160)	143 (137–150)	13 (7–18)	**0.001**
RHR	76 (72–80)	66 (61–71)	10 (6–14)	**<0.001**
	(Vigorous)	(Vigorous)		

Values are mean (95% confidence interval). HR: heart rate (bpm); RHR: reserve heart rate (%); RPE, rating of perceived exertion according to Borg CR-10 scale (A.U.). *Verbal descriptor of intensity according to the American College of Sports Medicine and Gunnar Borg. Bold p-values indicate significant results (*p* < 0.05).

**TABLE 3:** Heart rate recovery and handgrip strength after singles and doubles beach tennis session stratified by the competition level of the participants.

Variables	Level 1: Advanced (n = 11)	Level 2: Intermediate (n = 11)	∆	*p*-Value
**Heart rate**_**rec**_ **(bpm)**				
**Post_0′**				
Singles	156 ± 5 (150–166)*	142 ± 4 (133–150)	14 ± 7 (1–28)	**0.037**
Doubles	126 ± 8 (110–143)	138 ± 6 (126–151)	−12 ± 10 (−32 to 9)	0.254
**Post_1′**				
Singles	129 ± 4 (122–136)*	127 ± 3 (120–134)*	2 ± 5 (−8–12)	0.727
Doubles	114 ± 5 (105–123)	115 ± 4 (108–122)	−1 ± 5 (−13 to 10)	0.778
**Post_2′**				
Singles	119 ± 2 (115–123)	119 ± 3 (114–124)	0 ± 3 (−7 to 6)	0.897
Doubles	111 ± 4 (102–119)	119 ± 4 (111–127)	−8 ± 6 (−20 to 3)	0.169
**Post_3′**				
Singles	115 ± 4 (108–123)	112 ± 3 (107–117)	3 ± 5 (−6–12)	0.461
Doubles	108 ± 6 (92–119)	111 ± 3 (106–116)	−3 ± 6 (−16 to 9)	0.627
**Post_4′**				
Singles	112 ± 4 (104–119)	106 ± 2 (102–111)	6 ± 4 (−3–14)	0.234
Doubles	102 ± 6 (91–113)	108 ± 3 (102–113)	−6 ± 6 (−18 to 6)	0.326
**Post_5′**				
Singles	106 ± 4 98–114)*	102 ± 3 (97–108)	4 ± 5 (−6–14)	0.440
Doubles	94 ± 5 (84–104)	102 ± 3 (97–107)	−8 ± 6 (−19 to 3)	0.161
**Handgrip strength (KgF)**				
**Pre**				
Singles	43 ± 2 (39–48)	35 ± 1 (32–38)	8 ± 3 (3–14)	**0.002**
Doubles	41 ± 2 (37–44)	36 ± 1 (34–38)	5 ± 2 (0–9)	**0.034**
**Post 5′**				
Singles	42 ± 2 (38–46)	37 ± 1 (35–39)	5 ± 2 (1–10)	**0.018**
Doubles	43 ± 2 (39–47)	38 ± 1 (36–40)	5 ± 2 (1–10)	**0.024**
**Post 30′**				
Singles	42 ± 2 (38–46)	36 ± 1 (34–37)	6 ± 2 (1–10)	**0.010**
Doubles	43 ± 2 (39–48)	37 ± 1 (35–39)	6 ± 3 (1–11)	**0.020**

Values are mean ± standard error (95% Confidence Interval); *Singles is different from Doubles (*p* < 0.05); *p*-value is comparing the levels separately in the session (level 1 vs. level 2). Bold p-values indicate significant results (*p* < 0.05).

**TABLE 4:** Percentage of total match time spent in each intensity zone expressed as percentage of players’ maximal heart rate during singles or doubles beach tennis session stratified by the competition level of the participants.

Variables	Level 1: Advanced (n = 11)	Level 2: Intermediate (n = 11)	Δ	*p*-Value
**Heart rate**				
**Time <60%HRmax (%)**				
Singles	2.7 ± 0.7 (1.2–4.1)*	3.4 ± 1.4 (0.6–6.1)	0.7 ± 1.6 (−3.8 to 2.4)	0.658
Doubles	8.9 ± 1.9 (5.1–12.8)	6.4 ± 3.3 (−0.1–12.9)	2.5 ± 3.8 (−5.0–10.0)	0.514
**Time 61–70%HRmax (%)**				
Singles	8.1 ± 1.2 (5.6–10.5)*	8.2 ± 1.7 (4.8–11.6)	0.1 ± 2.1 (−4.3 to 4.1)	0.963
Doubles	26.8 ± 5.7 (15.7–38.0)	13.6 ± 4.1 (5.7–21.4)	13.2 ± 6.9 (−26.9 to 0.4)	0.057
**Time 71–80%HRmax (%)**				
Singles	13.6 ± 1.5 (10.7–16.5)*	18.7 ± 3.1 (12.6–24.9)*	5.1 ± 3.5 (−11.9 to 1.7)	0.138
Doubles	35.7 ± 3.1 (29.5–41.9)	25.0 ± 4.5 (16.2–33.8)	10.7 ± 5.5 (−21.5 to 0.8)	0.052
**Time 81–90%HRmax (%)**				
Singles	31.8 ± 3.3 (25.3–38.3)	32.0 ± 3.1 (25.9–38.1)	0.2 ± 4.6 (−9.1 to 8.8)	0.971
Doubles	21.9 ± 5.4 (11.4–32.6)	33.1 ± 4.9 (23.4–42.9)	11.2 ± 7.3 (−25.6 to 3.4)	0.129
**Time 91–100%HRmax (%)**				
Singles	44.0 ± 5.7 (32.8–55.2)*	38.0 ± 5.8 (26.6–49.4)*	6.0 ± 8.1 (−9.9–21.9)	0.462
Doubles	6.6 ± 3.6 (−0.4–13.6)	21.8 ± 6.5 (8.9–34.6)	15.2 ± 7.5 (−29.8 to −0.6)	**0.041**

Values are Means ± standard error (95% Confidence Interval); HRmax = maximal heart rate; *Singles is different from Doubles (*p* < 0.05); *p*-value is comparing the competition levels separately in the session (level 1 vs. level 2). Bold p-values indicate significant results (*p* < 0.05).

## 3 Results

The characteristics of the participants are shown on Table 1. Overall, they had healthy weight, were physically active (Thivel et al., 2018) and had over 3 years of experience playing the sport. The participants had similar characteristics, except for the training duration that was higher for advanced than intermediate players (*p* = 0.015). Temperature and relative humidity were similar for both sessions (singles: 13.7°C ± 3.5; 89.5% ± 7.3; doubles: 13.0°C ± 3.2; 92% ± 8.2). No adverse events occurred throughout the study.

No correlations were identified between the intrasession HR and RPE (after warm-up, and immediately after each set) during Singles and Doubles beach tennis sessions (*p* > 0.05 for all comparisons). Similarly, no correlation was found between the internal load and mean HR_reserve_ after Singles (*p* = 0.251) and Doubles (*p* = 0.395) beach tennis sessions.

### 3.1 Comparison between singles and doubles sessions

**FIGURE 1:**
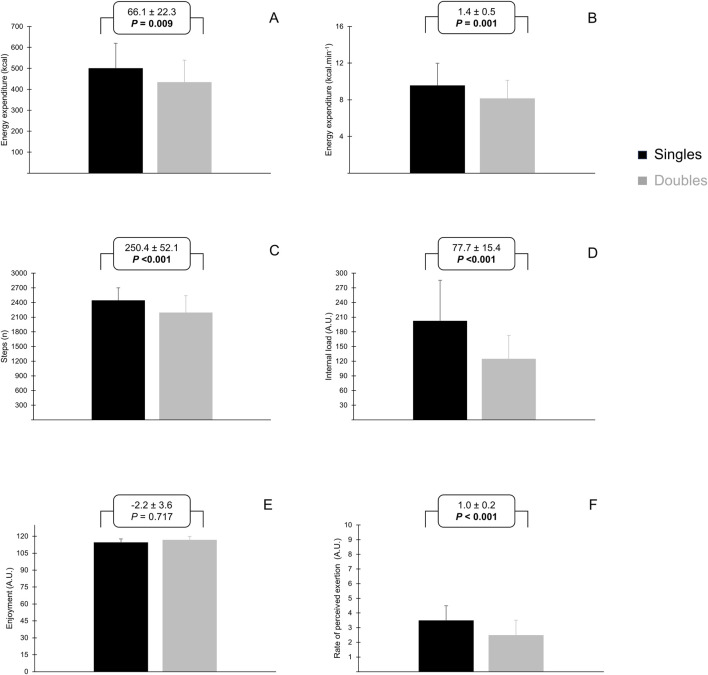
Absolute values of mean energy expenditure [panel **(A)**], energy expenditure per minute [panel **(B)**], number of steps [panel **(C)**], internal load [panel **(D)**], enjoyment [panel **(E)**], and rating of perceived exertion [panel **(F)**] during singles and doubles beach tennis sessions.

The mean and maximal HR loading, EE (total and by min), RPE, number of steps, the internal load, and the enjoyment level are presented in Figure 1 and Table 2. Overall, the values of these variables were higher in singles than doubles (*p* < 0.05), except for the enjoyment level that was similar for singles and doubles (*p* = 0.72).

**FIGURE 2:**
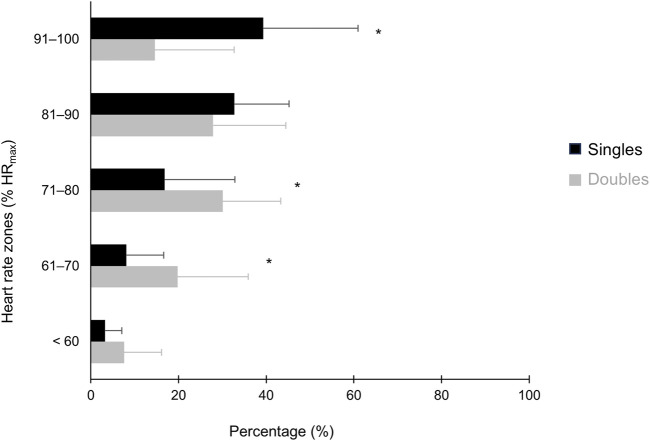
Percentage of total match time spent in each intensity zone expressed as percentage of players’ maximal heart rate during singles and doubles beach tennis sessions. *Singles different from doubles beach tennis session (*p* < 0.05).

Figure 2 shows the percentage of total time spent in each intensity zone expressed as percentage of players’ maximal heart rate (%HR_max_), with significant differences between singles and doubles at 61%–70%, 71%–80%, and 91%–100% HR_max_ (*p* < 0.05).

**FIGURE 3:**
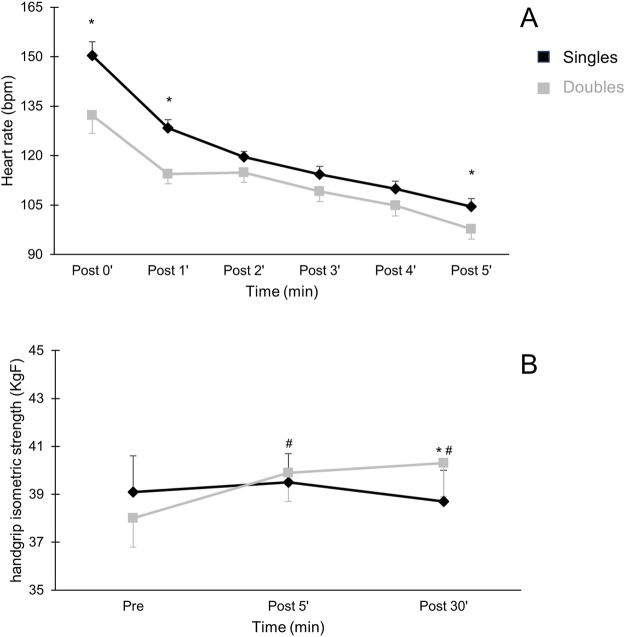
Recovery heart rate [panel **(A)**] and handgrip strength [panel **(B)**] after singles and doubles beach tennis sessions. *Singles different from doubles beach tennis session (*p* < 0.05). #Different from pre values of the same session (*p* < 0.05).

Figure 3 shows the recovery HR of the first 5 min after the sessions, and the maximal handgrip isometric strength assessed before, after 5′and 30′of the match. Recovery HR was higher after singles than doubles (Δ Time 0’: 18 ± 7 bpm, *p* = 0.013; 1’: 14 ± 4 bpm, *p* = 0.001 and 5’: 7 ± 3 bpm, *p* = 0.028). In relation to maximal handgrip isometric strength, the participants presented similar values before the sessions (Singles: 39/36 KgF; Doubles: 38/35 KgF). After the sessions, doubles presented higher handgrip strength in the dominant arm than singles at post 5'(Δ 2 KgF, *p* = 0.001).

### 3.2 Comparison between intermediate and advanced players

We ran additional analysis to explore potential differences between participants of different competition levels (Advanced and Intermediate players) during doubles (advanced vs. intermediate) and singles (advanced vs. intermediate) sessions (Tables 3, 4).

Significant differences were observed between the levels for the percentage of total time spent in each intensity zone. No significant differences were found for the number of steps, EE, RPE, mean HR, or maximal HR.

During the recovery period after exercise, advanced players exhibited a higher HR compared to intermediate players (*p* = 0.037) immediately after singles (post 0′). No other difference was found between competition levels for this variable.

Regarding isometric handgrip strength, advanced players consistently showed higher strength in the dominant arm compared to intermediate players at multiple time points (pre-exercise, post 5′and 30′ after the exercise session) after singles and doubles sessions.

The results of the percentage of total time spent in each intensity zone by level are shown in Table 4. In doubles, intermediate players spent more time at 91–100%HR_max_ than advanced players (*p* = 0.041).

## 4 Discussion

Beach tennis can be played individually (singles) or in pairs (doubles). In singles, the same player must cover the entire court and respond to every ball that crosses the net, potentially leading to different physiological demands compared with doubles, in which the workload is shared between two players. To the best of our knowledge, this is the first study to evaluate the physiological demands of beach tennis in women. Using a crossover trial, we also compared the physiological responses between singles and doubles matches, considering the potential influence of the training status of participants. The main results suggest that singles beach tennis induced a higher physiological loading than doubles, and participants of advanced categories may respond differently to those playing in the intermediate level. Moreover, independently of the competition level or characteristic of the match, a reduced RPE and a high level of enjoyment were achieved during the session. Taken together, our findings confirm the differences of physiological and perceptual demands of singles and doubles beach tennis matches and a possible influence of training status of participants in some but not all responses, which should be taken into account during the training organization and competitions of the modality for different populations such as patient groups or amateur athletes.

As a sport characterized by short intermittent bouts of small distance accelerations/decelerations, change of direction, and jumps on the unstable sandy surface, beach tennis players must react fast to always hit the ball before it touches the ground, resulting in a high stimulation of the cardiovascular system. Estimates of exercise intensity during a game may offer valuable information to optimize training methods, as well as to indicate how it may aid cardiovascular health of recreational practitioners. For example, in the well-research sport concept, Football Fitness, it has been shown that small-sided games can be modified to alter the exercise intensities, where altering the pitch size and/or number of players has great impact on the physiological loading (Randers et al., 2014b; Randers et al., 2014a). In the present study, relative and maximum HR were assessed to describe the cardiovascular strain of singles and doubles beach tennis games. When expressed as percentages of HR_reserve_ (i.e., %VO_2max_), an average of 76% for singles and 66% for doubles were found, being classified as vigorous and moderate physical activities, respectively (Garber et al., 2011). Players reached a peak HR loading of 181 ± 3 and 171 ± 3 bpm in singles and doubles, respectively, with values approaching maximum in singles. In addition, during singles matches players spent 39%, corresponding to 18 min of the total time in the 90–100%HR_max_ zone. In doubles, the HR value is considerably and significantly lower, with players spending only 15% or 7 min of the total time at this intensity. The HR loading values during beach tennis are comparable to women´s collegiate soccer (mean ∼75% HR_max_) (Jagim et al., 2020), tennis players (mean HR 128–164 bpm) (Cádiz Gallardo et al., 2023), and women´s recreational team handball players (mean 77%–79% HR_max_) (Pereira et al., 2023). Thus, it can be classified as an efficient method to provide a high HR loading, which is paramount for optimal improvement of cardiovascular health status (Batrakoulis et al., 2022).

The estimation of the metabolic responses through EE provides important information to the exercise prescription for both health and performance. We observed an EE of ∼8 kcal min^-1^ for doubles and ∼10 kcal min^-1^ for singles, with the EE of singles being ∼15% higher than doubles. These values are comparable to those achieved during traditional exercise modalities such as aerobic and combined exercises (Ferrari et al., 2018). Moreover, the number of steps was ∼10% higher for singles than doubles sessions. In fact, and in line with our primary hypothesis, we expected higher values when playing beach tennis individually, but it was uncertain how large this difference would be and how these values would be comparable to other types of physical activities. Grip strength influences game performance and also contributes to the prevention of musculoskeletal injuries (Elliott, 2006). In our study, the participants demonstrated mean grip strength values of ∼40 KgF in the dominant arm. Although the absence of studies assessing handgrip strength in beach tennis limits directs comparisons, it is noteworthy that our observed values surpassed those documented in a study involving female tennis athletes (18 years old) that presented mean grip strength of 33 KgF in the dominant arm (Pereira et al., 2011). We also assessed the handgrip strength 5 and 30 min after sessions to determine whether singles or doubles matches would result in forearm fatigue. We found no significant reduction of strength compared to baseline after singles. After doubles, we found an increase in handgrip strength when compared to baseline values and when compared to the singles session in the same period of time. We speculate that the local neuromuscular demand of singles may cause fatigue in the handgrip muscles because there is a necessity to activate it every action, while in doubles the demand is shared with the partner, allowing a better recovery between actions and reducing the local muscular fatigue.

The influence of training status on the physiological demands of beach tennis was also investigated in the present study. An important finding was that intermediate players spent more time at 91–100%HR_max_ zone than advanced players in doubles matches (22% *versus* 7% of total session’s time). Additionally, advanced players presented higher handgrip strength compared to intermediate in all time points (pre, post 5′and 30′). Taken together, these results suggest that advanced players are physically more conditioned than intermediates, highlighting the importance of physical fitness development to reach higher levels of competition. And confirms that beach tennis is a great training method for both levels and that players at intermediate level can also obtain high cardiovascular stimulation during the match. Future studies should explore the long-term training responses of recreational players of different competition levels.

The RPE values assessed during the exercise are directly associated with the physiological demand (i.e., HR) of traditional aerobic exercises (Cádiz Gallardo et al., 2023). The absence of correlation between these two variables during singles and doubles beach tennis suggests that RPE scale should be used with caution to control the exercise intensity of recreational sports. In fact, participants of recreational sports have to focus on elements of the game and seem to perceive less their effort (Carpes et al., 2021), and the high level of enjoyment during the practice (i.e., 91% and 93% for singles and doubles, respectively) may also contribute to the reduced perceived effort during exercise.

Some limitations should also be considered to properly interpret our findings. The convenience sample recruited in the study may present a possible selection bias and may not represent all the population who practice beach tennis, preventing us from the generalization of our findings to other levels such as beginners or professionals’ players. Besides, our sample consisted of women only, therefore limiting the generalization of our findings to the male population. The main strength of this study is its design, which included randomization and allocation concealment to reduce bias and ensure objective outcome assessment. We also employed standardized protocols and gold-standard measurements, such as HR monitors and accelerometers, to enhance the consistency and accuracy of the data collected.

## 5 Conclusion

Singles beach tennis resulted in higher physiological demands than doubles in women. Additionally, participants of different categories (intermediate *versus* advanced players) may respond differently in some but not all variables. Moreover, a reduced RPE during the matches and a high level of enjoyment after singles and doubles beach tennis were described by physically active women of different competition levels. These results may help coaches and beach tennis amateur players to better prepare for competitions, presenting beach tennis as an alternative to traditional exercises to improve physical fitness and cardiovascular health.

Beach tennis allows participants with chronic diseases or low physical and technical levels to practice the activity under the same rules, without the need to adapt the activity to make it attractive and effective for different populations. Other important advantages such as easy access to sand courts, the necessity of only 2–4 participants per match, allowing people of different age groups and levels of fitness/skills to play a sport that promotes pleasure and satisfaction during the activity, and lower risk of injury compared to traditional invasion sports (Kujala et al., 1995; Berardi et al., 2020), highlight the potential of beach tennis for both competitive and recreational purposes.

## Data Availability

The raw data supporting the conclusions of this article will be made available by the authors, without undue reservation.
